# Exploring the social-ecological potential for indigenous agroforestry in peri-urban areas: a participatory mapping approach

**DOI:** 10.1038/s41598-025-27864-3

**Published:** 2025-12-23

**Authors:** Mallika Sardeshpande, Tsitsi Bangira, Matilda Azong Cho, Trylee Nyasha Matongera, Tafadzwanashe Mabhaudhi

**Affiliations:** 1 Centre for Transformative Agricultural and Food Systems, University of KwaZulu-Natal, Scottsville, 3209 South Africa; 2 Department of Geography, School of Agriculture and Science, University of KwaZulu-Natal, Scottsville, 3209 South Africa; 3 Centre on Climate Change and Planetary Health, London School of Hygiene and Tropical Medicine, London, UK

**Keywords:** Agroecology, Farm design, Indigenous foods, Peri-urban agriculture, Suitability analysis, Agroecology, Urban ecology, Environmental sciences, Environmental social sciences

## Abstract

Peri-urban agroforestry can provide affordable, fresh, and nutritious food and a departure from conventional forms of cropping. Indigenous foods are well-adapted to local conditions, and may hold cultural and economic value for peri-urban residents. Social, ecological, and economic variables influence the feasibility of indigenous agroforestry in peri-urban areas. This study uses participatory mapping and geographic information systems (GIS) to assess these variables and to map suitable spaces and species for peri-urban indigenous agroforestry at three peri-urban sites in Durban, South Africa. We find that: land tenure, livelihood opportunities, and indigenous food perceptions factor into socioeconomic preferences; topography and soil quality influence ecological feasibility; access to water and roads influences perceived economic viability. Although GIS techniques can identify land suitability, participatory mapping adds local fine-scale context to enhance decision-making. Based on the social-ecological conditions at the three sites, we suggest specific configurations of locally adapted foods and farm designs for peri-urban agroforestry. Our study demonstrates how agroforestry is more feasible in places where basic living conditions are fulfilled, and how co-design can improve recognition of local needs, accessibility to services, and balancing urban green equity.

**Supplementary Information:**

The online version contains supplementary material available at 10.1038/s41598-025-27864-3.

## Introduction

Urban and peri-urban food production can improve the resilience of food systems in multiple ways^1^. From a logistic viewpoint, it can reduce the risk of supply chain failure and subsequent food and nutritional insecurity. Ecologically, it can reduce impacts from large-scale farming and transportation^2^, and socioeconomically, it can provide urban residents accessible, affordable, and nutritious alternatives to mass-produced and processed foods^3^. However, availability of land, labour, and materials are the main determinants of urban and peri-urban food production, in the global North and South^4^. In the face of densification and development, green space is a critical yet contested component of the urban landscape^5,6^. Cities worldwide have various legislations and allocations for food production in urban and peri-urban areas in communal gardens, private farms, and food forests^7^. These allocations help city planning to balance local economies, development interests, and urban environments, often in collaboration with local residents^8^. City-level adaptations are often crucial for successful implementation of national and regional food policies^9,10^.

Urban and peri-urban agriculture is an emerging and potent response to provisioning fresh and nutritious food, closing nutrient loops, creating circular economies, and reducing carbon footprints^11^. It can take various forms, from intensive indoor vertical farms, to communal agroecological (including agroforestry) spaces, with several intermediate configurations of social, ecological, and technological variables^12^. In this article, we focus on urban agroforestry as the proposed intervention to improve food and nutritional security among urban and peri-urban dwellers. Urban agroforestry systems, defined as urban landscapes combining crops and trees are increasingly recognised as productive landscapes with greater allied cultural and ecological benefits than conventional agriculture^13^. The cultural and recreational values associated with urban agroforestry systems can facilitate more equitable and widespread uptake of the food and nutritional produce yielded by these landscapes (ibid).

The feasibility of urban and peri-urban agroforestry could vary across different urban contexts. For example, in densely populated or historically established sections of cities, it could involve planting fruit trees and/or food crops along verges of transportation and utility lines that provide substantial nutrient yields^14–16^. In some cases, urban and peri-urban brownfields (previously developed land) may be reclaimed by municipalities or citizen collectives to grow food^17–19^. Urban parks and gardens established primarily for recreation may also be a significant and legitimate source of food and nutrition^20–22^. Structural constraints to urban and peri-urban agroforestry include the availability of contiguous land and arable soil^4,11^ and also resident and developer preferences for gentrified forms of nature, neighbourhoods, and greenspaces^23^. While biophysical and infrastructural variables can help determine suitability for urban agroforestry, social structures and perceptions are crucial to its long-term sustainability^24^.

Indigenous crops and trees are important components of agroecological systems. They are often resilient to local ecological stresses and shocks^25^, as well as human disturbance and extraction^26,27^. On farms, indigenous crops and trees provide pollination services, alternative income, and nutrition for farmers^28^. In urban and peri-urban areas, they can also provide habitat connectivity to wildlife^29,30^, including pollinators important to rural and urban food production^31^. This makes them ideal candidates for fragmented landscapes of high-intensity human use, such as urban and peri-urban areas, where large-scale farming is impractical. Foods from indigenous crops and trees are rich in high-quality micronutrients^32,33^, which are generally deficient in urban diets due to constrained accessibility and affordability. Recent research on indigenous crops has focussed on nutritional yields and land suitability for annual crops such as grains and tubers^34,35^. Although the potential of indigenous food-bearing tree species has been recognised^36–38^, the research and application of these in agroforestry is still nascent^114,115^. Therefore, in this study, we also attempt to identify the feasibility of planting indigenous crops and trees, comprising indigenous agroforestry, in urban and peri-urban areas, identifying synergies and constraints as applicable.

Participatory mapping was employed to document the cultural, economic, and social values of peri-urban agroforestry, seeking to establish its potential in enhancing livelihoods and promoting environmental sustainability. This involved mapping the variables favouring indigenous food production at three study sites. Thus, a suite of social science methods were used to elicit spatial and temporal data, trends, and preferences in landscapes and land uses^39^. The participatory mapping was conducted to promote democratic, inclusive, and locally appropriate decision-making when combined with GIS modelling techniques^40^. This is especially important in urban and peri-urban areas, where land use and land cover are fast-changing, and can often leave under-resourced communities impoverished^41^. People’s values for landscape features and uses can play an important role in successful landscape governance^42^, including the implementation and observance of regulations^43^. Peri-urban areas in the Global South differ from many in the Global North, in that the regulations and infrastructure in the former are not as organised and developed as urban areas^44^. This situation underscores the need for participatory mapping and GIS modelling to enable better planning and service provisioning in peri-urban areas in the Global South^45^. Our study follows a mixed methods approach giving equal importance to communities and experts in the mapping process^46^.

In this study, we seek to design locally appropriate indigenous agroforestry systems for urban and peri-urban areas with the aim of improving their food and nutritional security^47^. We combine data on social perceptions, spatial modelling, and indigenous agroforestry species to generate these designs, which are intended to inform local communities and municipal departments on feasible agroforestry and food security initiatives. Our study demonstrates how government policies and programmes, e.g.^55,56,115^ can be operationalised at local scale, e.g.^9,93,96,116^ by combining participatory research and different forms of knowledge. This study is part of the Durban Research Action Partnership between the local metropolitan eThekwini Municipality and University of KwaZulu-Natal, which aims to generate knowledge and learning to address the gap between scientific research, policy development and management within a local government^113^.

### The local context

As in the case of many developing nations, households in South Africa experience the triple burden of malnutrition, which includes undernutrition (stunting and wasting), micronutrient deficiencies (often termed hidden hunger), and overnutrition (overweight and obesity)^48^. Urbanising and westernising lifestyles influence the preference for cheap, convenient, ultra-processed and packaged food over traditional, nutritious, and fresh, diverse farm-based food^49^. Post-apartheid market liberalisation has facilitated the penetration of cheap and calorie-dense low-nutrient foods into local markets for consumers and incentivised the export of high-quality foods such as fruit and vegetables to foreign markets for producers^50^. Smallholder farmers who cannot export or sell to mainstream domestic markets often struggle with a lack of infrastructure and institutional support to improve yields and sales^51^.

In the broader socioeconomic sense, unemployment and economic inequality result in income poverty and food poverty, and limited opportunities for people experiencing poverty to engage in either primary production or secondary activities to secure an income^52^. Particularly in cities, legacy spatial planning also constrains access to greenfields (previously undeveloped land) and greenspace, which can often be a source of food or materials to support the household economy^37,53^ As a result of apartheid-era policies, green infrastructure and public access to it is well-developed in affluent neighbourhoods, but is severely lacking in poorer (often racially differentiated) neighbourhoods^54^. Allocating and enriching urban and peri-urban spaces for food production are a priority on the National Development Plan for South Africa^55^, and could also contribute to national-level cross-cutting initiatives like the Integrated Food Security and Nutrition Programme and the Natural Resources Management Programme^56^. In this study three communities in the peri-urban areas of the eThekwini Metropolitan Municipality (that houses Durban, hereafter eThekwini) were consulted to identify spaces where food production can be undertaken, to enhance food and nutritional security.

## Methods

### Conceptual framing

**Fig. 1:**
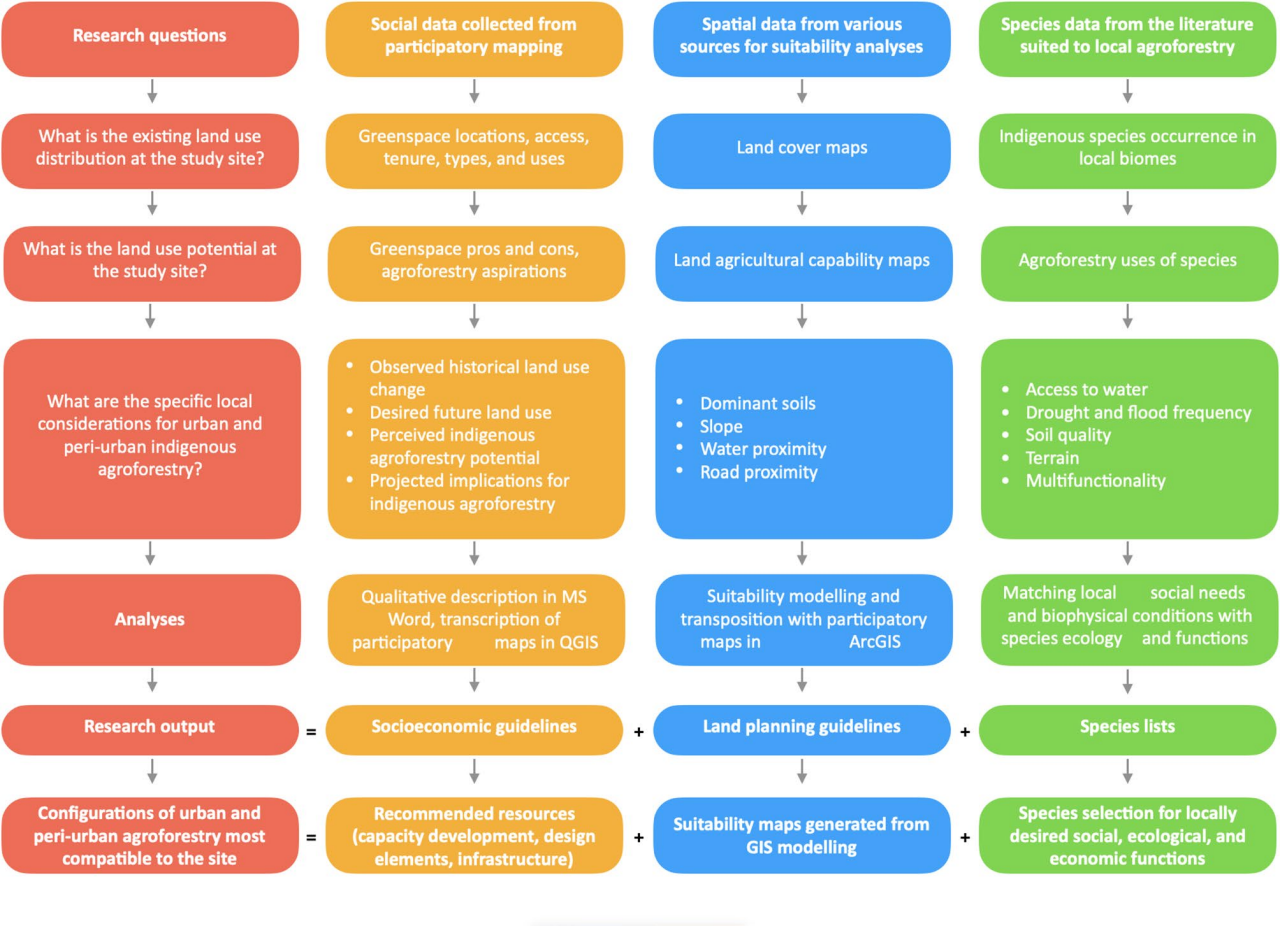
Conceptual framing of the study, research questions, data collected, and analyses.

The study aimed to identify the most compatible configurations of peri-urban food production given the social-ecological conditions at each site. The study used an overarching landscape ecology approach^57^ to define the landscape configuration, land use, land cover change, and landscape management guidelines. The study combined participatory mapping and GIS suitability analyses to identify suitable areas for peri-urban food production. The research objective was achieved by answering three questions (Fig. 1) in both the participatory mapping and suitability analysis approaches. The methodology characterised local social-ecological factors and existing and potential land use for food production at each site (Fig. 1). These were analysed to produce socioeconomic and land use guidelines suggesting configurations of peri-urban food production suitable to each site. Landscape configuration was determined using spatial datasets (Table 1). The land use and cover change were determined from social data, to account for socio-polictical nuance about these changes. The management guidelines emerging from the analysis of these datasets were combined to propose locally appropriate landscape management guidelines that included biophysical suitability maps, social aspirations and needs. The conceptual framing and resulting management guidelines resonate with recent calls for agriculture to incorporate indigenous species as ‘trade-ons’, through appropriate design for ‘land maxing’^20,114^. The management guidelines resulting from this study are applicable examples of mainstreaming biodiversity and indigenous knowledge into productive and adaptive peri-urban agroforestry^115^.

**Table 1:** Influence percentage for each factor used to produce the final suitability maps.

Geographic factor	Description	% of influence
Land cover	The 2020 South African Land Cover data was acquired from the South African Department of Forestry, Fisheries and the Environment online portal, publicly available at https://egis.environment.gov.za/data_egis/data_download/current. Seven broad land cover classes, namely built-up areas, forests (commercial and natural), grasslands, water bodies, fallow lands, agricultural lands and bare areas, were identified in the three areas	35
Agricultural land capability	The land capability data, defined as the total suitability for usage without causing harm to grazing, woods, wildlife, and crops that require frequent tillage, was acquired from the Department of Agriculture, Land Reform and Rural Development. The eight-class land capability classification constitutes the basis of most subsequent attempts at bringing land capability concepts under measurable parameters (Online Appendix Table A3)	20
Dominant soils	The soil data was obtained from the Soil and Terrain Database (SOTER), freely accessible at https://www.isric.org/explore. Four major soil categories were identified at the three study sites (Online Appendix Table A2)	15
Slope	The slope percentage map was derived from the Copernicus Digital Elevation Model (COP-DEM) with a 30 m spatial resolution using the slope function in ArcGIS Pro software. The slope map was divided into ten equal interval classes, according to^74^, to represent levels of slope suitability for the expansion of peri-urban agriculture	15
Proximity to water sources	To help irrigate peri-urban food production, the study considered the distance to the available water sources. Rivers were the only available water sources that were considered in this analysis. The river networks were extracted from a 30 m COPERNICUS- DEM using the watershed module in R Studio^75^. ArcGIS Pro software used The Euclidian distance function to generate distance maps from the existing rivers	10
Proximity to the main road	For accessibility to the farmlands and easy transportation of harvest to the nearby markets, the distance from the roads was incorporated in the analysis. The road network data was acquired from the Department of Transport online portal, accessible at https://gis1.kzntransport.gov.za/arcgisportal/home/. ArcGIS Pro software used The Euclidian distance function to generate distance maps from the existing roads	5

### Study area

**Fig. 2:**
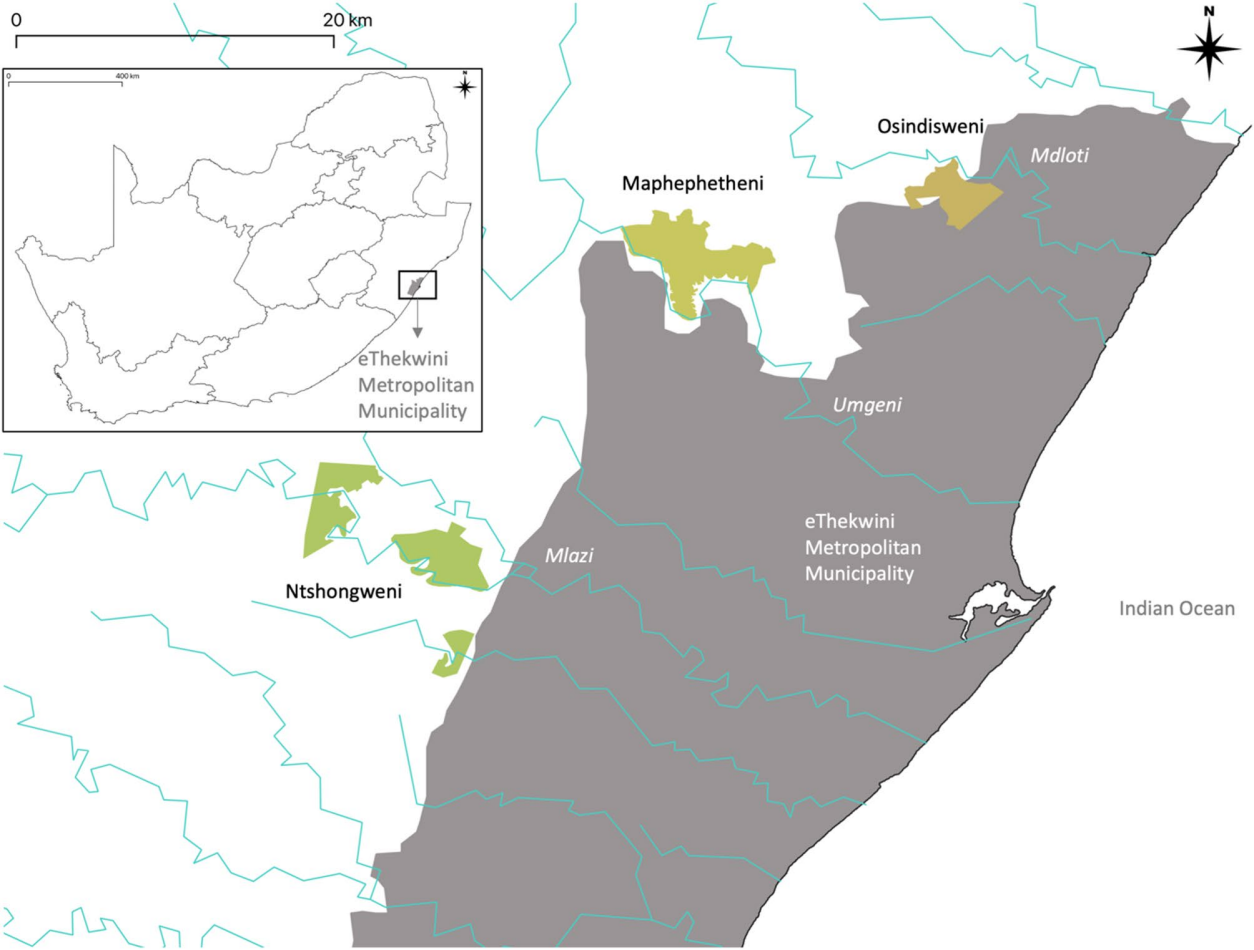
Location of study sites in the local context and South Africa (inset). Some sites are fragmented because of dual land tenure—traditional and municipal.

The eThekwini municipality is host to a population of 3.9 million people, in its urban centre of Durban, as well as several peri-urban areas^58^. Due to legacy planning and diverse tenure systems, the city centre and suburbs have designated greenspace, whereas the peri-urban areas are more informal and sporadic in structure. About 44% of the land in eThekwini falls under the Ingonyama Trust, governed by traditional chiefs, and is not subject to the same planning requirements as municipal land^37^. The Durban Metropolitan Open Space System (DMOSS) was instituted in the 1990s to plan and govern land use across formal, informal, protected, and indigenous greenspace in urban and peri-urban areas^59–61^. Under this system, land use is restricted in areas of ecological importance, ecological restoration offsets are required where feasible, and urban greening and agroforestry are promoted in collaboration with municipal departments and NGOs^62^. The municipality routinely undertakes reforestation and restoration across these open spaces, with the dual intention of improving biodiversity and supporting local bio-economy livelihoods^63^. There is also a strong emphasis on removing and controlling invasive alien species and planting endemic and indigenous species in greenspaces^37^. Given this background, our research questions consider the diversity of land tenure and indigenous species, especially trees, that are of interest at each study site (Fig. 2). The three study sites were suggested by eThekwini Municipality as areas of interest for rolling out peri-urban agroforestry interventions, as part of their municipal agroecology programme^9,116^.

- Maphephetheni: situated on the mountainous area surrounding the Inanda reservoir on the Umgeni River, built in the 1980s, contiguous with the suburb of Inanda. The peri-urban settlement consists of savanna and grassland vegetation. Areas degraded by invasive species (e.g. *Acacia* spp.) and fire are being replanted with useful food and forage species (e.g. *Canthium* spp., *Ficus* spp., *Searsia* spp.) to encourage sustainable land use^64^. The municipality’s erstwhile Environmental Planning and Climate Protection Department (EPCPD), now the Biodiversity Management Department, engages members within these communities to nurture saplings for restoration, increase awareness and stewardship and prevent cyclical degradation and restoration. The area has a population density of 344 persons per km^2^ as per the 2011 census.
- Ntshongweni: situated along the ridge of the Shongweni dam on the Mlazi River, built in the 1920s, with vegetation consisting of riparian forest, grassland, and some wetland. Rail and road connections to the urban centres of Durban and Pietermaritzburg have attracted investment in transport and logistics centres in the vicinity, and more recently, in commercial retail and residential development. The EPCPD also runs invasive alien control and reforestation programmes at Ntshongweni. The area has a population density of 399 persons per km^2^ as per the 2011 census.
- Osindisweni: situated along the ridge of the Hazelmere dam on the Mdloti River, built in the 1970s, with vegetation consisting of riparian forest and grassland. It is adjacent to Buffelsdraai, a site historically degraded by intensive sugarcane farming, and currently serving (since 2008) as a suburban landfill ring-fenced by indigenous forest fragments^65^. These fragments are gradually expanded and connected by ongoing planting, and although most of the forest is protected, the periphery and a small section of the site have been earmarked to grow indigenous food-bearing tree species for surrounding communities. The EPCPD does not yet run restoration programmes at Osindisweni. The area has a population density of 439 persons per km^2^ as per the 2011 census.

### Participatory mapping workshops

One participatory mapping workshop was conducted in each community between September 2021 and March 2022. Local chiefs and councillors were approached for their consent to engage with the community, and for assistance in recruiting community members to participate in the workshops. We acknowledge that this recruitment strategy may have resulted in a representational bias, but assert that we communicated to each chief and councillor the need to engage with all sections of the community including youth, elders, employed, unemployed, and women. The aim of the research was introduced at the beginning, and informed consent was obtained from all participants to record their responses and take photographs for research purposes only. The study was ethically reviewed and approved by the Humanities and Social Sciences Research Ethics Committee of the University of KwaZulu-Natal in June 2021 (Protocol Reference Number HSS/1971/017D). All methods were performed in accordance with the Economic and Social Research Council guidelines on ethical scientific research.

The outline map (Online Appendix Fig. A1) of the community with key features, namely, rivers, roads, schools, and hospitals, was presented to the participants. They were asked: (i) What are the various greenspaces in the community, and what are their tenure and access terms? (ii) What are the resources and uses associated with each of these greenspaces? (iii) What are the positive and negative characteristics of these greenspaces? (iv) What changes have these greenspaces undergone in the past 10 years? (v) What changes, if any, would the community like to see in these greenspaces? (vi) What species of food, especially indigenous trees, grow or are grown in the community, and where? (vii) What food species would the community want growing in their greenspaces, and where? We used the most open and commonly accepted definition of greenspace, implying undeveloped land that harbours some form (cultivated or wild) of vegetation, and is used for one or more of the purposes of: agriculture and food cultivation, cultural and recreational activities, foraging, fishing, and grazing^66^.

A native isiZulu speaker interpreted the questions and responses, and all responses were recorded on the map during the discussion. Names of places and indigenous plants were recorded in isiZulu. “Tree” spaces were recorded as a separate category overlapping with other types of spaces. They included home gardens, sports fields, and open spaces, as they may be fragmented yet productive in their food and non-food yields (e.g. fibre, fuel, medicine, wood). Food production was recorded as a use only when explicitly mentioned by participants (e.g. home gardens or open spaces where food was gathered from plants but not grown as crops were not deemed used for food production). The data collected were analysed qualitatively using reflexive grounded theory, e.g.^67^ for emergent themes and descriptions in MS Word. Quotes from participants were anonymised using the monikers ‘Respondent n’, and presented to illustrate examples, claims, and arguments.

### Species selection

Social-ecological attributes of land use and cover change at each site were derived from the data shared by respondents. Landscape design configurations were suggested in response to these attributes, with functions such as biophysical tolerance and cultural importance. The landscape management guidelines recommended biome-appropriate indigenous food species from^36,68^, and^69^. The underlying philosophy of this species allocation was to increase land productivity for local food and nutritional security, regenerating natural capital for human and environmental health, and enabling local communities to generate human, social, physical and financial capital through the planting of useful indigenous species^114^.

### Suitability analyses

Several factors influence land suitability for urban agricultural farming. Biophysical, socio-economic, and technical aspects are some of the primary factors. The principal purpose of land suitability for urban crop farming is to predict the potential and limitation of land for crop production^70^. Generally, determining suitable areas for crop farming in urban areas revolves around making the most sustainable use of land resources while avoiding depleting other resources^71^. Crop farming land suitability analysis requires an efficient decision support system to analyse and interpret the related ecological, environmental and spatial information. GIS and participatory GIS are combined with multicriteria decision analysis (MCDA) methods to deliver a better spatial decision^72^.

This study determined suitable areas for peri-urban food production in three stages. First, the factors affecting the agricultural uses were set up as criterion maps. Secondly, all the factors were scored in the suitability range based on expert opinion and the results from the participatory mapping workshops. Finally, GIS spatial analysis modelling techniques were used to generate suitability maps for the three sites.

The study adopted six factors, as suggested by^73^, to set up criterion maps. The factors include land cover, agricultural land capability, dominant soils, slope, proximity to water sources and proximity to the main road. The weighted overlay in ArcGIS Pro was used to generate the final suitability maps based on the percentage of influence for each geographic factor. Here, the influence of each factor (weights) was arbitrarily chosen based on the results of the interviews and experts’ knowledge. Thus, each layer contributes to the influence based on the type of agricultural land use (Table 1). In this study, the final suitability maps were reclassified into five classes with suitability scales ranging from highly suitable to not suitable.

## Results and discussion

### Characterising greenspace attributes, perceptions, preferences and potential for peri-urban agroforestry through participatory mapping

The workshops lasted about 90 min at each site, and involved between 11 and 29 participants. Participants included but were not limited to, representatives of the ward, workers with different departments of the municipality such as community services, education, environment, and health and sanitation, local smallholder farmers, part-time employed and unemployed youth and elders, private sector employees, and representatives of local NGOs, churches, and cooperatives. The participation in the workshops was variable and limited due to the ongoing Covid-19 restrictions at the time. Nevertheless, we believe the depth and diversity of the discussions are representative of the sites.

Current land use of greenspaces included provisioning and recreation, although the former was reported as significant only at Maphephetheni and Ntshongweni (Table 2). Greenspaces are an important avenue for urban and peri-urban foraging at all three sites, providing residents with resources and recreational opportunities^76,77^. Food production was intentionally undertaken in communal and public greenspaces at the aforementioned sites, but not at Osindisweni. Tenure over greenspaces also varied across the sites, and areas under the traditional authority, i.e. the local chief, were used for recreation and grazing, but not to grow food. Land tenure is an important driver of land use and stewardship, and traditional authority tenure can deter land-based livelihoods such as food production and agroforestry. For example, lack of accountability and definition in spatial allocation in communal areas can result in violation of land use agreements^78^, reducing certainty of long-term land use, and subsequent investment of labour and capital in food production^79^. It may also result in rent appropriation by powerful stakeholders at the expense of the community^80^ and undemocratic development on land intended for food production, especially in urban and peri-urban areas^81^. This may partly explain why at Osindisweni, where most greenspaces are under communal tenure, participants expressed low interest in food production and agroforestry.

**Table 2:** Existing land use: types of greenspaces, their tenure, and uses at the three study sites. Species information is listed in Online Appendix Table A1.

Types of space	Number	Tenure	Uses	Resources	Remarks
Maphephetheni					
Communal areas	2	Chief	Grazing, recreation	Fodder, fruits (banana, guava)	Beautiful views, but far away, and harbour monkeys
Open spaces	3	Public	Grazing, recreation	Fish, fruits (mango + 10 spp.), herbs (watercress, wild spinaches)	Includes reservoir fishing, plantation, “nature” areas
Food gardens	4	Neighbourhood	Food production	Vegetables (carrot, spinach)	Low yielding
“Tree” spaces	3	Private and public	Food production and recreation	Fruits (13 spp.)	Includes sports field, household, riverine trees, and plantation gumtrees
Ntshongweni					
Open spaces	3	Public	Food production, fuelwood collection, grazing, recreation	Fish, fodder, fuelwood, vegetables (beetroot, cabbage, carrot, chilli, onion, peppers, spinach)	Includes reservoir fishing and erstwhile sports field that turned into a wetland and now stands protected
Food gardens	5	Cooperatives	Food production	Maize, vegetables (see above)	Need to know what to grow, when, where, and how
“Tree” spaces	3	Private and public	Food production and recreation	Fruits (11 spp.), fuelwood	Includes household and riverine trees, and an erstwhile game reserve that “no one uses now”
Osindisweni					
Communal areas	4	Chief	Recreation	Fruits (8 spp.), scavenged food and goods from landfill	Includes bush, open space, landfill buffer, and riverine; fruits only collected in riverine, which is far away
Food gardens	1	School	Unused	None	Poor upkeep
“Tree” spaces	5	Private and public	Food production and recreation	Fruits (8 spp. + litchi to purchase), vegetables to purchase	Includes household and riverine trees, an agricultural hub, and a litchi farm

The productivity of greenspaces varied across sites, with Ntshongweni residents earning and saving money from the sale of food produced in home and public greenspaces (Table 3). Participants at Ntshongweni expressed an interest in diversifying their food production by including indigenous crop and animal species.

**Table 3:** Perceived land use potential and food production feasibility in greenspaces at the three study sites.

Greenspace characteristics	Maphephetheni	Ntshongweni	Osindisweni
Positive aspects	Experience of “nature” and “views”; recreational activities such as fishing, hunting, picnicking, and swimming with family		
	Use of communal areas for grazing and foraging for food and wood; food gardens contributing to community nutrition		
		Savings and earnings from local food sales from cooperative gardening	
Negative aspects	Recreational greenspaces are located far away from residential areas	Privatisation of resource reserve and lack of community access	Conversion of area to landfill and conservation buffer; poor waste management and invasive control; ecological, health, and safety impacts
	Lack of access to seeds, water, fencing, and food garden training hinders food production		
Changes over the past decade	Gradual: trees are now outnumbered by households within the community	Gradual: dwindling of game animals; Drastic: appearance of a wetland at a sports field (and subsequent municipal protection); Recently, numerous food garden cooperatives were formed during the coronavirus pandemic	Gradual: conversion and expansion of the landfill resulting in indiscriminate waste dumping and scavenging in the buffer area around the community; Drastic: fumes and frequent fires from the landfill pose significant respiratory problems
Future prospects	Prioritise the ‘One home one garden’ concept, wherein each household can grow food in their yards through material and technical input	Open the Reserve to the public for food production (orchards), forestry (fuelwood), or community facilities (school, youth centre, or disaster high ground); plant indigenous trees as a replacement for invasive aliens and crop shade	Demand better greenspace management through invasive alien control and waste collection; prioritise public infrastructure like schools, soup kitchens, shops, and health centres over food gardens and other greenspaces
Food production potential	Low-yielding food gardens, fishing, foraging, and livestock supplement the food economy; high-yielding home gardens	High-yielding crop and food gardens; high-yielding home gardens; need knowledge and training on locally suited species of food plants and animal breeds for agroecology	Perceived unviability of air and soil due to waste dumping, landfill fires; poor upkeep of existing food garden
Tree planting feasibility	Accessible to most people, valued for food, fodder, air and temperature regulation, and wood for poles; preference for mainstream foods such as avocado, oranges, and peaches over indigenous species	Accessible and valued for food, fodder, shade, and rainwater sequestration; placement of trees to prevent damage to property during floods and mudslides	Limited access to household trees, which are “ancient, our own” and valued by the community; planting more trees may not be feasible until the landfill pollution problem is alleviated

“The municipality [representative] tells us that there is a market for indigenous crops and chickens. We would like to learn about how to farm these so that we can sell not just within our communities, but also to the urban market.”—Respondent 1.

Natural greenspaces were “far away” for residents of Maphephetheni and Osindisweni.

“[That place] is far away, so we visit only on some weekends, maybe once or twice a year. When we go there, it is with family and friends. We can take our time and be one with nature.”—Respondent 2.

These observations make a case for the development of more accessible parks and gardens for residents closer to residential areas. Planning for such should consider local perceptions of safety and environmental quality to minimise unintended consequences such as dereliction or gentrification^82^. Across all three sites, lack of plant material, stable water supply, livestock predation, and know-how were reported as hindrances to food production in community food and school gardens.

“There are times in the summer when we don’t have water [on tap] for some ten, twenty days. This is when the plants also need water, and we also need [drinking] water. That’s why our [community food] gardens are not successful. The crops die.”—Respondent 3.

“What we need to know is how to grow crops and trees properly. Both common and indigenous ones. We need to learn how to water them care for them, how to harvest them at the right time.”—Respondent 4.

Participants made different site-specific recommendations to improve food productivity. For example, in Maphephetheni, home gardens were considered more effective than public gardens, as protecting them from water shortages, flooding, and livestock and human predation was easier. On the other hand, Osindisweni respondents prioritised shops and soup kitchens as means to improve food security, as they believed their land to be no longer viable for food production due to pollution associated with the landfill.

“We live close to the city. We do not need to grow our own food. What we need is more shops to buy our food from. We need schools and soup kitchens to support our people with meals for food security. This is the support we need from the government.”—Respondent 5.

“The soil here is very degraded. There is so much dumping, so many fires. People suffer from respiratory problems because of this environment. Crops and trees will never grow here. If the municipality wants to help us, they should collect our garbage more regularly.”—Respondent 6.

### Suggesting locally occurring, useful indigenous species suited to respective site attributes for peri-urban agroforestry

Based on the pros, cons, and potential identified in the previous stages, we characterise seven site attributes and six response functions that can be served by greening for urban food production, in addition to improving food and nutritional security (Table 4). We suggest using thorny plants as fencing structures to prevent livestock predation while simultaneously maintaining biomass for humans and non-humans in the form of fruits and fodder. Given the use of greenspaces for non-food and non-timber products and the need for invasive alien replacement, indigenous trees with multiple uses can be planted in various greenspaces. Some of these species already grow in greenspaces across these sites (Table A1) but were not referred to as serving the proposed functions. None of the herbs or crops were specifically mentioned by participants during the elicitation at the workshops.

**Table 4:** Design configurations based on synthesised site attributes, desired response functions, reviewed literature on indigenous food species, and participatory mapping locations, for Maphephetheni (M), Ntshongweni (N), and Osindisweni (O). (Y = Yes, N = No, indicating species suitability at site).

Site attributes	Response functions	Indigenous species	Latin name	Location	M	N	O
Livestock grazing	Live fence fruits	Kei apple	*Dovyalis caffra*	Food gardens Home gardens School gardens	Y	Y	Y
		Numnum	*Carissa sp.*		Y	Y	Y
		Monkey orange	*Strychnos sp.*		Y	Y	Y
Use of biomass for fencing, fodder, food, and medicine, and replacement of invasive aliens	Multipurpose fruit trees	Dune currant	*Searsia natalensis*	Any open spaces	Y	Y	Y
		Marula	*Sclerocarya birrea*		Y	N	N
		Milkwood	*Sideroxylon inerme*		Y	Y	N
		Brown ivory	*Berchemia discolor*		Y	N	N
		Red ivory	*Berchemia zeyheri*		Y	Y	Y
		Sour plum	*Ximenia caffra*		Y	Y	Y
		Turkey berry	*Canthium inerme*		Y	Y	N
		Wild medlar	*Vangueria infausta*		Y	Y	N
		Wild plum	*Harpephyllum caffrum*		N	Y	Y
Slope (M, N) Communal land tenure, low labour availability (O)	Fast-growing annual herbs	Amaranth	*Amaranth sp.*	Food gardens School gardens Any open spaces	Y	N	Y
		Jute mallow	*Corchorus olitorius*		Y	Y	N
		Nightshade	*Solanum nigrum*		Y	Y	N
		Spider flower	*Cleome gynandra*		Y	N	Y
		Wild mustard	*Rapistrum rugosum*		N	N	Y
Flood risk	Waterlogging-tolerant crops	Sweet potato	*Ipomoea batatas*	Food gardens Along slopes	Y	Y	N
		Taro	*Colocasia esculenta*		Y	Y	N
Indigenous crop inclination (N)	Indigenous grains	Sorghum	*Sorghum bicolor*	Flat open spaces	N	Y	N
		Teff	*Eragrostis teff*		N	Y	N
Poor soil quality (O)	Annual nitrogen-fixing crops	Bambara groundnut	*Vigna subterranea*	School gardens Flat open spaces	N	Y	Y
		Cowpea	*Vigna unguiculata*		N	Y	Y
		Pigeon pea	*Cajanus cajan*		N	Y	Y

We acknowledge that cultivation of some of the trees and crops suggested in Table 4 may require significant investment in technical training and infrastructure. For example, *Carissa*, *Dovyalis*, and *Harpephyllum* are dioecious species, requiring careful selection and planting of sufficient male and female plants in close proximity to ensure fruiting. Cultivation of crops, especially grains, may require knowledge of seed accessions, and access to postharvest facilities for processing and storage^110–112^. The local-scale matching of trees and crops to sites undertaken in this study is validated by species distribution based on biophysical parameters^35,37^. Findings from our research present the first step towards operationalising national policy on indigenous knowledge and agriculture^115^, and further directions for developing the required physical and social infrastructure by the local municipality.

A number of these indigenous trees serve as a significant conduit to the intergenerational transfer of ecological knowledge and a connection to nature^69^, which in turn forms an important part of biocultural diversity and landscape stewardship^83^. Fast-growing herbs that require little input can be grown in marginal areas where the terrain poses difficulties, or where land tenure induces uncertainty. Crops that can resist waterlogging, enrich soil, and improve local productivity are also suggested where appropriate. Similarly, choices of crops exist for areas that are more prone to drought or heatwaves, or for marginal soils or shaded or windy areas^68^. Spatiotemporal intercropping of these with conventional crops can help remediate soil^84,85^. Surplus production of indigenous crops and trees can feed into short and high value supply chains to urban centres, e.g.^38,86^.

### Determining biophysical land suitability for peri-urban agroforestry using geospatial analyses

**Fig. 3:**
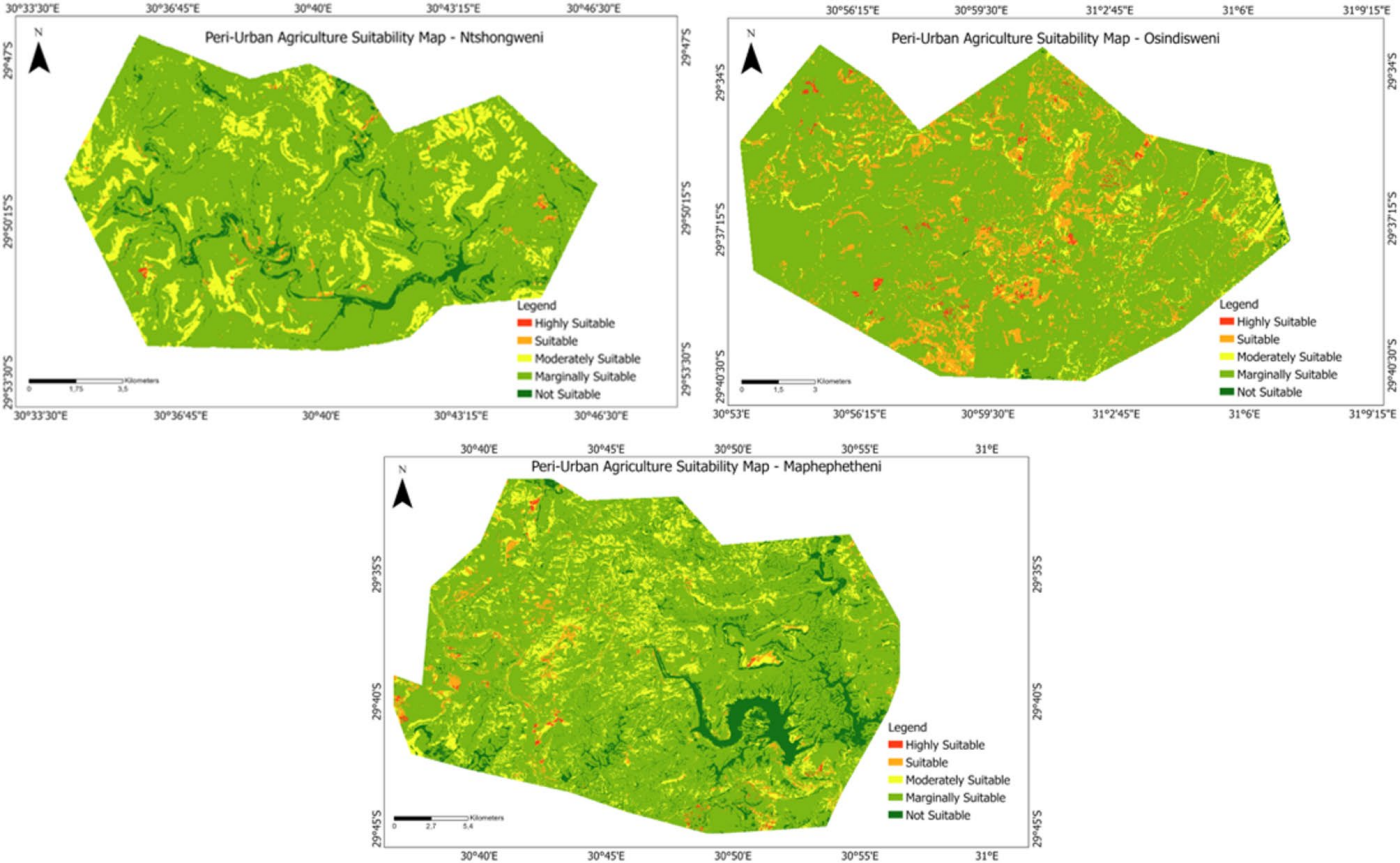
Peri-urban agriculture suitability maps for (**a**) Osindisweni, (**b**) Ntshongweni and (**c**) Maphephetheni.

Figure 3 shows the maps produced using expert-derived weights and value functions in each area. According to experts` knowledge, a higher weight was suggested for land cover than for agricultural land capability, dominant soils, slope, proximity to water sources and proximity to the main road. It should be noted that bare land plays a major role in delineating suitable urban areas for food production. Based on the results, a final weight of 0.35 was assigned to land cover. The final suitability maps for each area were divided into five agriculture suitability quality classes defined at discrete levels, allowing for comparisons between the three maps. The classes include suitable, moderately suitable, marginally suitable and not suitable areas for peri-urban agriculture. A simple visual comparison of the suitability patterns revealed by the three maps shows that Osindisweni has the greatest proportion of highly suitable and suitable areas for peri-urban agriculture. The Osindisweni area has suitable areas such as land cover, agricultural suitability, and open spaces, which favour the area’s suitability for agriculture. Also, this area has a good road and river network.

**Fig. 4:**
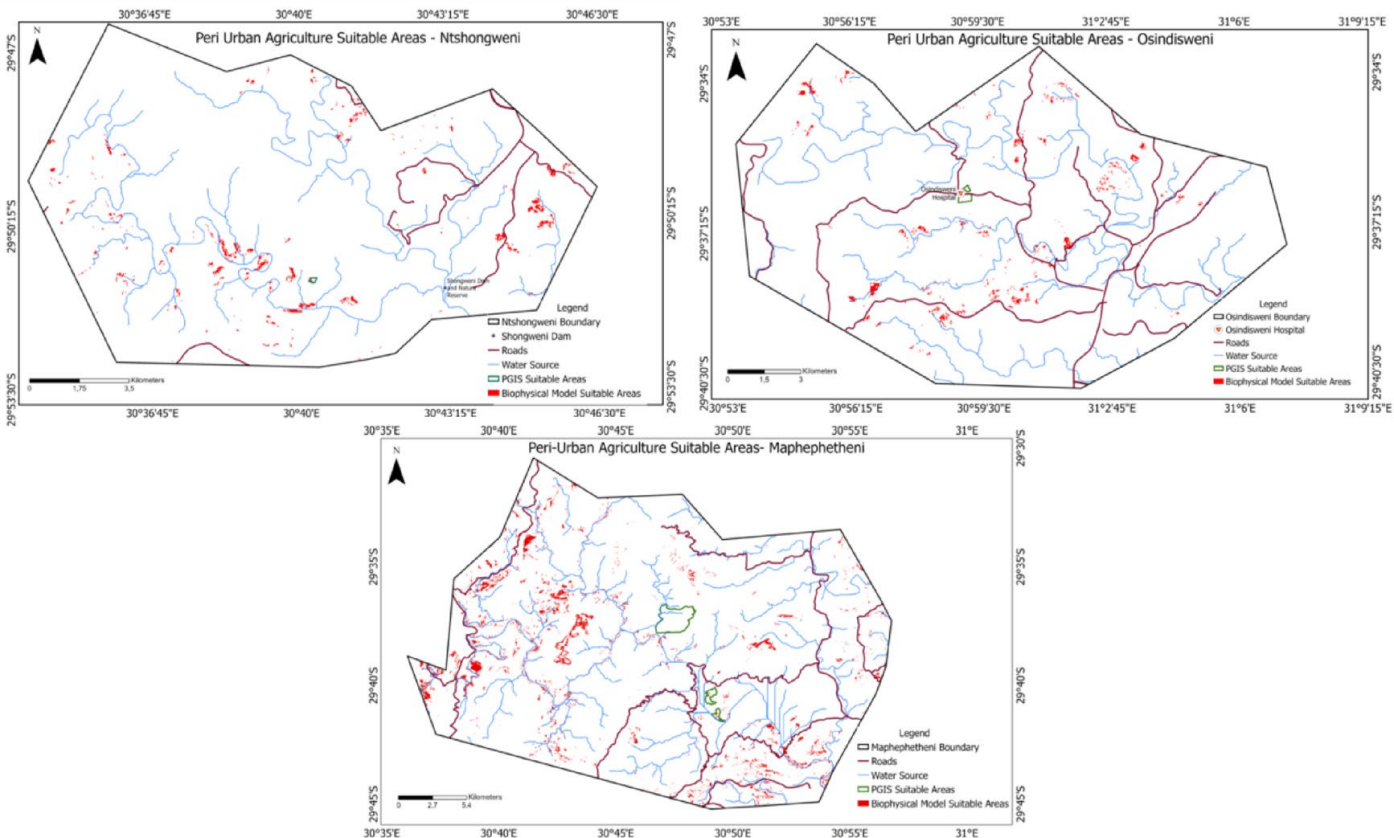
The suitable areas for peri-urban agriculture after overlaying both the PGIS and GIS layers in Osindisweni, Maphephetheni and Ntshongweni districts.

For further analysis, the highly suitable, suitable and moderately Suitable areas were combined and overlaid with PGIS-identified suitable areas. Areas identified in the participatory mapping workshops tended to overlap with the high- to moderately-suitable classes of land identified in the GIS (biophysical) model (Fig. 4). This shows an agreement between the two methods used in this study to identify areas suitable for peri-urban food production at the three sites. Using both approaches strengthens the estimates of suitable areas by identifying the areas for which both approaches identify while minimising the number of wrongly identified areas. These maps will significantly value future land use and land cover change analysis for urban crop production.

Table 5 shows that 75% of the study area in Maphephetheni, 4.53% in Ntshongweni and 0.21% in Osindisweni is permanently unsuitable for peri-urban crop production. These areas have unsuitable land cover and steep slopes, far from the road and river network. With a 10.74% suitability rate, the Osindisweni area has the highest potential for peri-urban food production, followed by Maphephetheni (1.2%) and Ntshongweni (0.84%). Generally, a small portion of the total area in all three study areas is suitable for urban crop production. For successful and effective peri-urban food production, growing crops with high production over a small piece of land, such as onions, herbs, garlic and leaf vegetables, is advisable.

**Table 5:** The distribution of land suitability for each site from the PGIS land suitability analysis model.

Site	Maphephetheni		Ntshongweni		Osindisweni	
	Area (km^2^)	Percentage	Area (km^2^)	Percentage	Area (km^2^)	Percentage
Highly suitable	0.23	0.13	0.28	0.17	1.7	0.78
Suitable	2.2	1.2	1.4	0.84	23.4	10.74
Moderately suitable	11.3	6.15	23.63	14.1	12.8	5.87
Marginally suitable	32.2	15.52	134.7	80.37	179.6	82.4
Not suitable	137.9	75.01	7.6	4.53	0.45	0.21

Statistics comparing the number of cells assigned to each suitability class for the three maps are presented in Table 5 and Fig. 3. The difference between the areas of the site was up to 50 km^2^, with Ntshongweni being the smallest (167.61 km sq.), followed by Maphephetheni (183.83 km sq.) and Osindisweni (217.95 km sq.). The GIS suitability analysis indicated that Osindisweni has the largest absolute area of suitable land and the largest ratio of suitable to unsuitable land, with over 99% of its area being suitable for greening for food (Table 5). Conversely, Maphephetheni had the smallest suitable land area, accounting for about 25% of its total area. Ntshongweni also had a high ratio of 96% of its land suitable for greening for food.

Osindisweni’s proximity to erstwhile sugarcane fields^65^ corroborates the finding that it has a greater proportion of suitable to marginally suitable agricultural land. However, more recent social-political developments, such as rapid urbanisation and the expansion of landfills have resulted in food production being perceived as untenable in the area. Indeed, expanding industrial and urban activities can accelerate a shift from land-based livelihoods and a decline in soil and water quality^87,88^. Our findings reiterate the importance of triangulating land use planning across large to fine scales through participatory methods. Alongside soil depth and nutrients, the slope is an important landscape determinant of land suitability for conventional food production^89^. Notwithstanding, results from our participatory and synthesis process offer options to reinforce local food and nutritional security through innovative design elements^12^. Despite the proximity to water sources, last-mile connectivity to arable land emerged as a significant limitation for agroforestry at the three sites. Plans to promote food production should consider strategies to manage nutrient flows in soil and water^90,91^. Excess runoff of agricultural enrichment materials may threaten water quality and safety. This is especially important given the immediate dependence of peri-urban and urban dwellers on surface and groundwater^92^. Agroecological strategies, including organic and circular inputs, are likely to alleviate environmental and food safety issues^93^. We acknowledge that our model considers arable land suitability in general, but that this may vary depending upon crop and tree species. Future research on multi-species indigenous agroforestry could be more species-specific, e.g.^94^.

## Limitations

Our study has two main limitations, namely the sample frame, and analytical depth. Our strategy to enlist participatory mapping workshop participants relied mainly on the local ward councillors and traditional chiefs, as this is standard practice to demonstrate respect of local authorities and build trust with local communities in the area. Participatory mapping work was carried out during a period when pandemic lockdown restrictions were in effect to varying degrees. Under these conditions, the participation in workshops was variable across the three sites, and it is possible that certain sections of communities that are less socially empowered may have been under-represented in the sample. Secondly, the scope of this study was to combine basic social, spatial, and species data to suggest locally appropriate peri-urban agroforestry design. Therefore, detailed evaluations of equitable access, agroclimatic variability, agronomic feasibility, etc. were not possible at this stage. We suggest that future research can delve into these specificities at site and regional scale. We posit that findings from this study provide a valuable baseline for research and implementation.

## Policy, practice, and research implications

This study highlights the role of participatory co-design in developing urban agriculture configurations. It combines a social-ecological systems lens^95^ with a landscape ecology approach^57^ to derive locally appropriate designs using locally adapted species. The communities expressed their aspirations for local food and nutrition security, which took on different forms. The participatory mapping outcomes demonstrate how local social-ecological and political situations influence preferences and feasibility of urban food production. Where food production is ongoing, diversification is welcomed, but where basic living conditions such as water supply and environmental quality are compromised, food production becomes secondary to expectations of urban living standards. This reiterates the need for participatory planning in the development of urban agriculture as a sustainable and citizen-driven enterprise in South Africa^96^. Our study demonstrates an interdisciplinary and participatory design approach to designing urban green infrastructure for ecosystem services^97^. Co-design has the benefits of recognising local needs, making services more accessible, balancing environmental regulatory frameworks with land use guidelines, and improving local resilience for urban green equity^98,99^. Further work in these communities has included the planting of a community agroforestry trial^100^ and an agroecology demonstration hub^101^ using indigenous species. These sites will serve as living learning laboratories for indigenous urban and peri-urban agroforestry. Communities will be involved in research related to assessing biodiversity and ecological implications such as species richness and plant biomass^102,103^, and also with development of market linkages for urban and peri-urban agroforestry, e.g.^24,114^.

## Conclusion

This study finds that while GIS tools can generate detailed information on land use suitability, the participatory process allows for the democratic exchange of knowledge, particularly in fast-changing socioeconomic landscapes like peri-urban areas. Specifically, the site where people have secure land tenure, service delivery, and existing involvement in agroforestry was found to be more suited to diversified multifunctional agroforestry configurations. Conversely, the site with uncertain land tenure and service delivery could support fewer configurations despite having the most are of suitable land according to GIS modelling. The site with most area of non-suitable land could also be matched with a high number of agroforestry configurations. This integrated approach can aid the development of site-specific solutions, forming dynamic governance co-produced by communities and institutions^8,83^. The participatory research component also helps to build an adaptive, responsive community of practice around each site by engaging with relevant stakeholders in non-political terms^104–106^. Through innovative design considerations, our findings aim to enable synergistic improvements in food and nutritional security, agroecology, and multifunctional urban green infrastructure^107,108^. The outputs contribute to achieving the sustainable development goals (SDGs) 2 (reducing hunger), 3 (promoting health and wellbeing), 9 (infrastructure innovation), 11 (sustainable cities and communities), 12 (responsible consumption and production), 13 (climate action), 15 (life on land), and 17 (partnerships) (SDG 2015)^109^. Policymakers and planners can draw from this partnership template using participatory research to feed into programmatic implementation at local scale^8–10,113–116^. In turn, participatory action research can be a conduit to intervention scaling, and to generating social-ecological evidence through living laboratories. This form of exchange is especially applicable to developing the field of indigenous local ecological knowledge^28,35,38,86,94,115^.

## Supplementary Information

Below is the link to the electronic supplementary material.

Supplementary Material 1

## Data Availability

All data generated or analysed during this study are included in this published article [and its supplementary information files].
