# Supplementary material for: Exploring the social-ecological potential for indigenous agroforestry in peri-urban areas: a participatory mapping approach
Source: Sci Rep. 2025 Dec 23;15:44344. doi: 10.1038/s41598-025-27864-3 (PMC12728215; doi:10.1038/s41598-025-27864-3)
Supplement: Supplementary file 1 — Supplementary Material 1 [file 41598_2025_27864_MOESM1_ESM.pdf]

Supplementary Material for:

Sardeshpande, M., Bangira, T., Matongera, T. N., Azong Cho, M., & Mabhaudhi, T. (2025). Exploring the social-ecological potential for indigenous agroforestry in peri-urban areas: a participatory mapping approach. *Scientific Reports*.

Figure A1: Outline maps (as displayed to the communities for participatory mapping) and mapped greenspaces (handwritten text and shapes replaced with typed text and GIS-referened polygons).

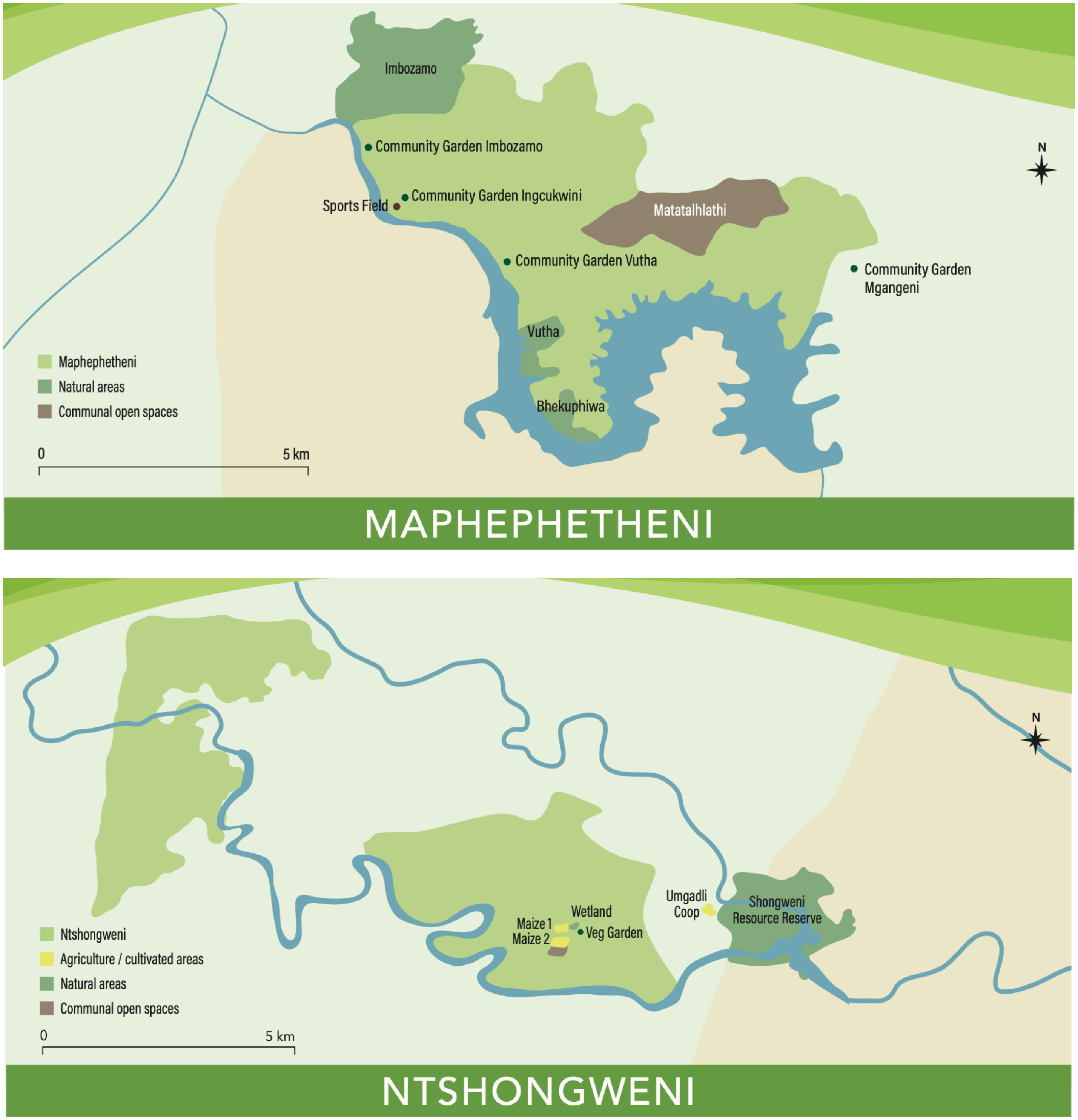

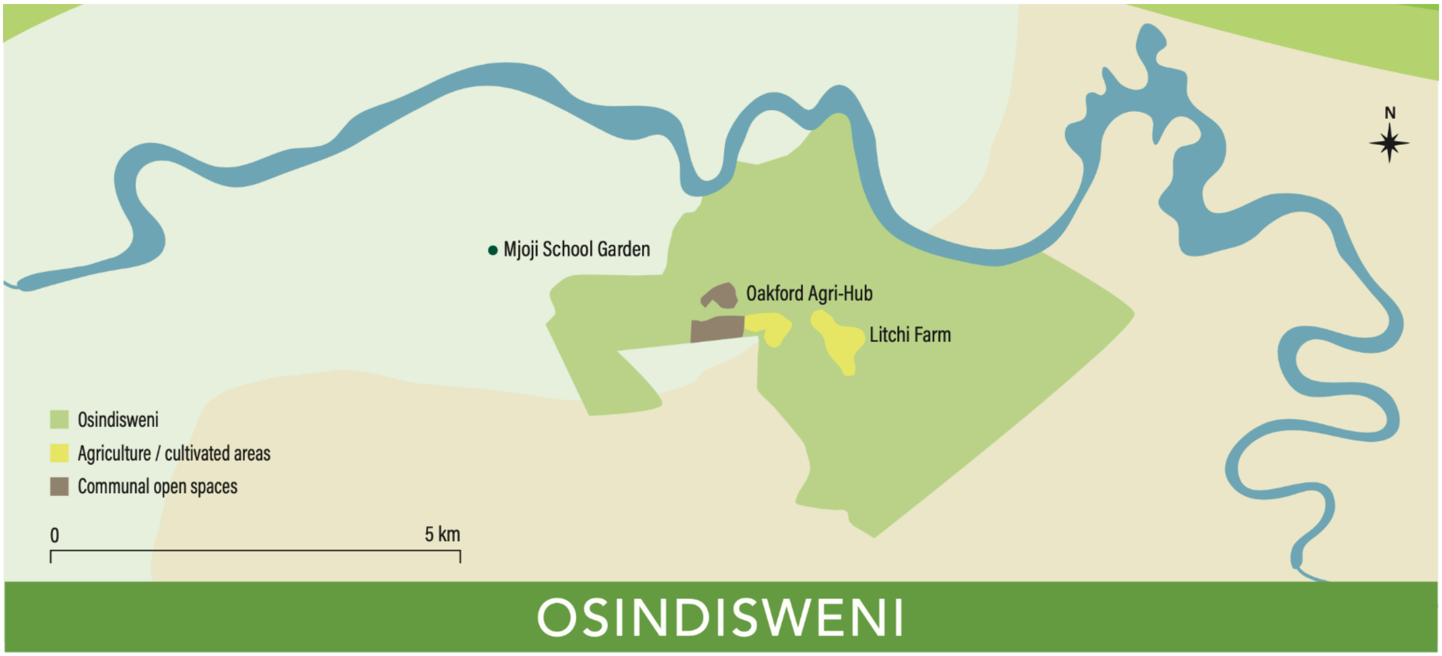

OSINDISWENI

Table A1: List of fruit tree species reported at Maphephetheni (M), Ntshongweni (N), and Osindisweni (O), with * indicating indigenous species

#	Scientific name	Common name	isiZulu name	M	N	O
1	Canthium inerme	Turkeyberry*	iMvuthwamini	■		■
2	Carissa sp.	Numnum*	aMatangulu		■	
3	Ficus sur	Boom cluster fig*	umKhiwane	■		
4	Harpephyllum caffrum	Wildplum*	umGwenya	■		
5	Morus sp.	Mulberry*	amaJigjolo	■	■	■
6	Sclerocarya birrea	Marula*	uMganu	■	■	■
7	Searsia rehmanniana	Blunt-leaved currant*	isiHlakothi	■		
8	Syzigium sp.	Waterberry*	uMdoni	■	■	■
9	Trichilia emetica	Natal mahogany*	umKhuhlu	■		
10	Vangueria infausta	African medlar*	umTulwa	■	■	■
11	Ximenia caffra	Sourplum*	umTunduluka	■		
12	Carica papaya	Pawpaw	=		■	
13	Citrus limon	Lemon	=		■	
14	Litchi chinensis	Litchi	=			■
15	Mangifera sp.	Mango	=	■	■	■
16	Musca domestica	Banana	=	■	■	
17	Persea americana	Avocado	=		■	
18	Prunus persica	Peach	=		■	■
19	Psidium guajava	Guava	=	■		■

Table A2: Soil categories descriptions

Soil Category	Characteristics	Reference
Haplic Acrisols (ACh)	Inherently infertile soils, that are quickly chemically and organically degraded when utilized.	(Tho & Hoa 2017)
Dystric Regosols (RGd)	Characterized by shallow, medium- to fine-textured, unconsolidated parent material of alluvial origin and the absence of a significant soil horizon formation due to dry or cold weather events. Regosols are used mostly for irrigation farming or low volume grazing.	(Hansson et al. 2011)
Dystric Leptosols (LPd)	Soils with a very shallow profile depth (indicating little influence of soil-forming processes), and they often contain large amounts of gravel.	(Anikwe 2000)
Haplic Phaeozems (PHh)	Porous, fertile soils suitable for intensive cropping High organic matter content, well aerated soils with moderate to strong structures.	(Bobrovskii 2010)

Table A3: Land capability groups and land use options

Land use capability class	Land use options	Land capability groups
I<br>II<br>III<br>IV	W F LG MG IG LC MC IC VIC<br>W F LG MG IG LC MC IC<br>W F LG MG IG LC MC<br>W F LG MG IG LC	Arable land
V<br>VI<br>VII	W F LG MG<br>W F LG MG<br>W F LG	Grazing
VIII	W	Wildlife
	W – Wildlife<br>F – Forestry<br>L – Light Grazing<br>MG – Moderate Grazing<br>IG – Intensive Grazing<br>LC - Poorly adapted cultivation<br>MC – Moderately well adapted cultivation<br>IC – Intensive, well adapted cultivation<br>VIC – Very intensive, well adapted cultivation	
